# Automatic Rectification of the Hybrid Stereo Vision System

**DOI:** 10.3390/s18103355

**Published:** 2018-10-08

**Authors:** Chengtao Cai, Bing Fan, Xin Liang, Qidan Zhu

**Affiliations:** Department of Automation, 4139, Building No. 61, Harbin Engineering University, Nan Tong No. 145, Harbin 150001, China; caichengtao@hrbeu.edu.cn (C.C.); liang941219@hrbeu.edu.cn (X.L.); zhuqidan@hrbeu.edu.cn (Q.Z.)

**Keywords:** hybrid stereo vision, projection model, automatic rectification

## Abstract

By combining the advantages of 360-degree field of view cameras and the high resolution of conventional cameras, the hybrid stereo vision system could be widely used in surveillance. As the relative position of the two cameras is not constant over time, its automatic rectification is highly desirable when adopting a hybrid stereo vision system for practical use. In this work, we provide a method for rectifying the dynamic hybrid stereo vision system automatically. A perspective projection model is proposed to reduce the computation complexity of the hybrid stereoscopic 3D reconstruction. The rectification transformation is calculated by solving a nonlinear constrained optimization problem for a given set of corresponding point pairs. The experimental results demonstrate the accuracy and effectiveness of the proposed method.

## 1. Introduction

Stereo vision systems have been widely used in tasks such as surveillance [1], search [2], exploration [3], autonomous navigation [4], mapping [5], and obstacle detection [6] for their ability to recover 3D information of real-world scenes. Additional advantages can be derived using omnidirectional cameras in stereo vision systems [7]. These cameras allow the horizontal field of view to be increased to 360 degrees. Although imaging models and camera calibration methods for different types of catadioptric mirrors have been studied [8,9], these cameras have very limited resolution and are unable to provide close observation of particular targets. To improve the applicability of omnidirectional cameras, camera networks consisting of catadioptric and perspective sensing devices [10,11,12] have been proposed. The combination of these two cameras helps to continuously monitor the entire surveillance area while making detailed observations of specific targets. The hybrid stereo vision system combines the advantage of the 360-degree field of view cameras with high-resolution imaging from the conventional cameras, but it also poses challenges for epipolar geometry and stereo rectification. Omnidirectional images cannot be processed by the pinhole imaging model of conventional images [13], meaning that there is no unified imaging model for omnidirectional and conventional images. Consequently, the geometry of the traditional stereo vision system [14,15,16] cannot be applied to the hybrid stereo vision system.

Generally, there are two types of methods to solve the aforementioned problem. One is to perform external calibration using the specific relation between the camera pairs or a large amount of known corresponding points. Under a local planar assumption, a non-linear approach for registering images in a hybrid vision system without requiring the calibration of cameras is proposed in [17]. When the position of the omnidirectional camera and the conventional camera are fixed, a checkerboard pattern with two different colors is used to acquire the geometric relation of the stereo camera system with heterogeneous lenses [18]. A geometric relation between the omnidirectional camera and the conventional camera is derived using manually obtained corresponding points [19]. Several calibration image pairs are obtained under known spatial positions. The extrinsic parameters are extracted via re-projecting known corresponding 2D points into a 3D space [20]. Three different models have been proposed to obtain fundamental matrices for hybrid vision [21]. Although these methods can achieve accurate results, they are limited by the high computational complexity or the priors of the relative position of the hybrid cameras.

Another method is to use pan-tilt-zoom (PTZ) cameras instead of conventional ones in the hybrid vision system, where it is not necessary to calculate the extrinsic parameters explicitly. Spatial mapping can be easily computed between an omnidirectional camera and a PTZ camera. Specifically, the pan-tilt angle of the PTZ camera is acquired by the corresponding points in the omnidirectional camera when the system is operating. This approach assumes that the pan-tilt angles are extremely correlated with the corresponding points. In some studies [12,22], 3D reconstruction is based on data collection and neural network fitting, but in other studies, the assumption of geometry constraints is made. For instance, in [23,24,25], it is assumed that the omnidirectional camera and PTZ camera are coaxial, or even that they share a common origin [26]. However, these assumptions are violated when the optical axes of both omnidirectional and PTZ cameras do not coincide. In addition, the corresponding points may not be in the same 2D plane without calibration. These problems will significantly affect the accuracy of 3D reconstruction.

In practical applications, for example, a hybrid vision system can be used for target tracking and observation tasks. The conventional camera will move as the position of the target changes to ensure that the target is within the common field of view of the omnidirectional camera and conventional camera. When the hybrid vision system is used in surveillance scenarios, the conventional camera will move as the monitored area changes. Therefore, automatic stereo rectification is highly desirable, while the accuracy is also needed. 

In our study, an automatic stereo rectification approach for one omnidirectional camera and one conventional camera is proposed. Compared with state-of-the-art methods [20,27], the main contributions of the proposed approach are as follows:

- A perspective projection model is proposed for the omnidirectional image, which significantly reduces the computational complexity of 3D formulation for mixed-view pairs.
- A method based on a novel, well-defined cost function for optimizing the normalization matrix is employed, which can calculate the rectification transformation more accurately.
- To evaluate the performance of the proposed automatic rectification method and to provide a direct application, a target tracking and odometry hybrid vision system is established based on an automatic rectification approach.

The remainder of this paper is organized as follows: The proposed automatic rectification approach for hybrid stereo vision system is presented in Section 2. Section 3 describes the configuration of the hybrid imaging system. The methodology, including the novel perspective projection model for the omnidirectional image and the method for optimizing the normalization matrix, is described in Section 4. In Section 5, the experimental results are presented, and a direct application of our rectification method is also provided. In Section 6, we discuss our results, limitations and future work. The conclusion is given in Section 7.

## 2. Proposed Automatic Rectification Approach

**Figure 1:**
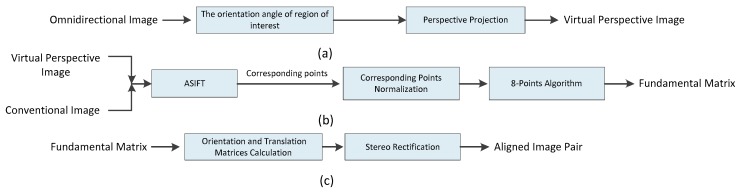
Block diagram of the proposed automatic rectification approach. (**a**) Acquisition of the virtual perspective image. (**b**) Calculation of the fundamental matrix. (**c**) Stereo rectification.

The block diagram of automatic stereo rectification for hybrid vision is shown in Figure 1. The proposed approach consists of three parts, including acquisition of the virtual perspective image in Figure 1a, calculation of the fundamental matrix in Figure 1b, and stereo rectification in Figure 1c.

Figure 1a shows how we calculated the direction angle α of the region of interest in the omnidirectional camera, after which the region of interest was projected onto a virtual plane using the proposed projection model.

In Figure 1b, the conventional camera is shown to rotate α degrees to ensure that the common field of view of the two cameras is the region of interest. Affine scale invariant feature transform (ASIFT) [28] was used to extract and match the features in the virtual perspective image and conventional image, after which the proposed optimization method for normalizing corresponding points was applied. Finally, the fundamental matrix was calculated by means of the 8-point algorithm [29].

Figure 1c shows how the fundamental matrix was decomposed into rotation and translation matrices of the virtual perspective image and conventional image. Finally, aligned image pairs could be obtained by remapping.

The main processing modules will be described in detail in the following sections.

## 3. Hybrid Omnidirectional and Conventional Imaging System

**Figure 2:**
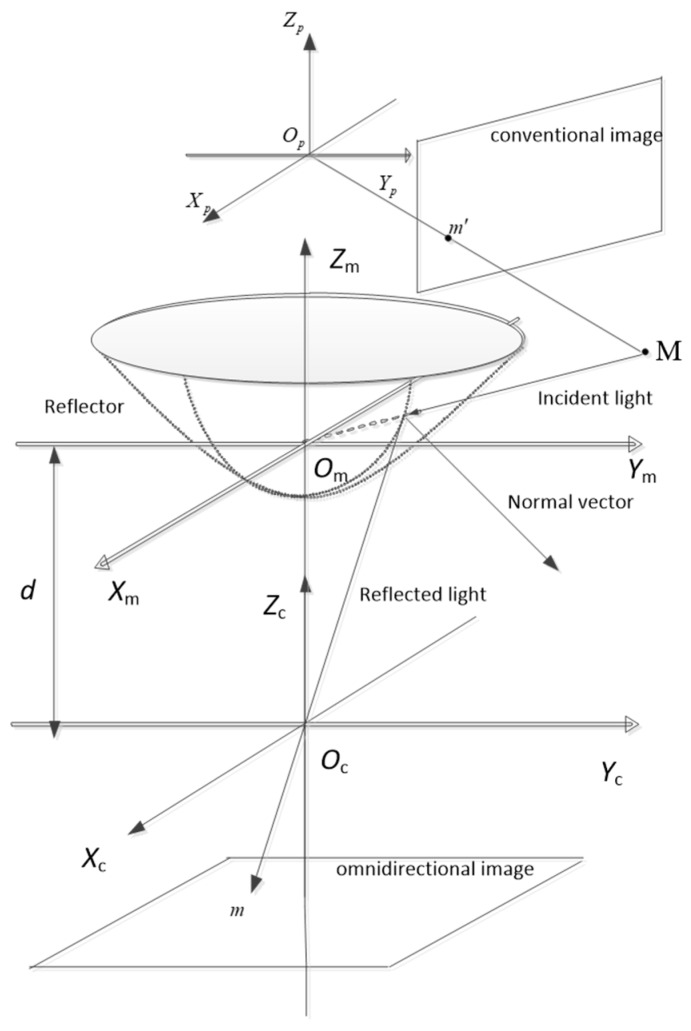
The configuration of the hybrid vision system. It consists of a perspective camera and a catadioptric camera with a hyperboloidal mirror.

The configuration of the hybrid vision system in this paper is shown in Figure 2. It illustrates the point correspondence relation between an omnidirectional image and a conventional image. The hyperbolical mirror was chosen for the omnidirectional camera to ensure that it had a single effective viewpoint, which is a necessary condition for the generation of pure perspective images from the captured images. We placed the omnidirectional camera vertically with the conventional one. The vertical installation not only avoids the own occlusion of the system, but also has a large common field of view. There are three coordinates in the hybrid vision configuration—conventional camera coordinate $X_{p}Y_{p}Z_{p}$, catadioptric coordinate $X_{m}Y_{m}Z_{m}$, and omnidirectional coordinate $X_{c}Y_{c}Z_{c}$. The projection center of the two cameras is $O_{p}$, and $O_{c}$. d is the distance between the catadioptric coordinate $X_{m}Y_{m}Z_{m}$ and projection center $O_{c}$. $d=2\sqrt{a^{2}+b^{2}}$. a and b are the long and short axes of the hyperbolic mirror, respectively. The 3D point M was projected to point to m of the conventional image plane by linear mapping. It also projected to point $m^{\prime }$ of the omnidirectional image through the incident and reflected rays, which is nonlinear mapping. If the extrinsic parameters of the two cameras are known, the 3D point *M* can be determined uniquely by m and $m^{\prime }$.

## 4. Methodology

### 4.1. Virtual Image Generation

To avoid the complex geometric relationship between the omnidirectional image and the conventional image, a novel perspective projection model for the omnidirectional image is proposed in this section. Unlike the conventional image, the generation of a virtual perspective image from an omnidirectional image is not one-to-one linear mapping—in this case, a simple perspective projection model is desirable.

**Figure 3:**
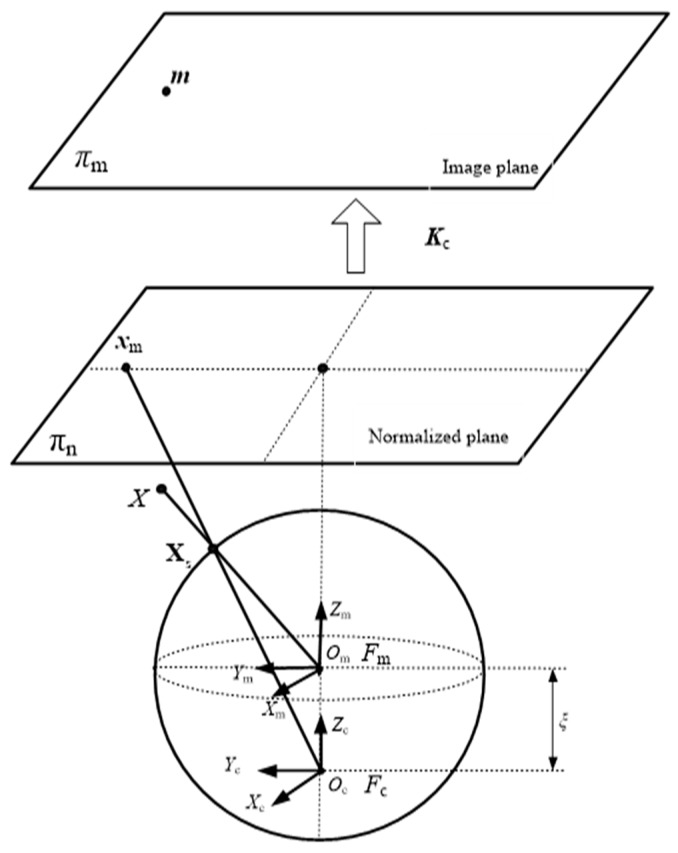
The unit sphere model for the catadioptric camera.

As described in [30], a central catadioptric projection is equivalent to two-step mapping via the unit sphere. As shown in Figure 3, $O_{m}$ is the origin of the catadioptric coordinate and $O_{c}$ is the origin of the camera coordinate. For a general omnidirectional camera, the optical axis was aligned to the line defined by $O_{m}$ and $O_{c}$. Point X in the 3D coordinate was projected onto a unit sphere located at the origin of the catadioptric coordinate, $O_{m}$. In the coordinate of the sphere, $X_{s}={\left[X_{s},Y_{s},Z_{s},1\right]}^{T}$. Then, the projection of X on the normalized plane could be given by:

(1) $$x_{m}=(X_{s},Y_{s},Z_{s}+\xi )\;$$

where ξ∈[0,1] is the distance between $O_{c}$ and $O_{m}$. Therefore, the corresponding point in the omnidirectional image plane could be obtained by:

(2) $$m=K_{c}x_{m}\;$$

where $K_{c}$ is the intrinsic parameter of an omnidirectional camera. According to this, we were able to perform a back projection from the omnidirectional image and then reproject it onto a virtual plane.

**Figure 4:**
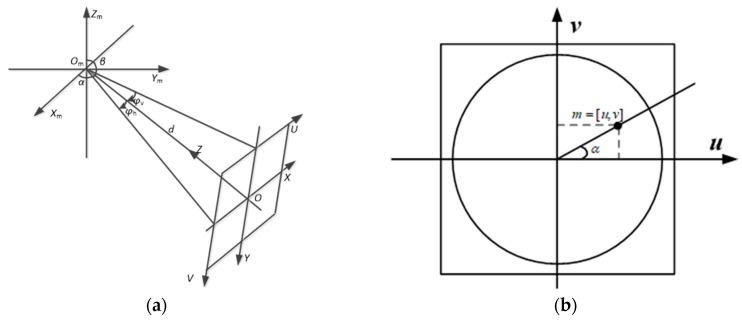
(**a**) The effective viewpoint of the virtual perspective image; (**b**) the coordinate of the omnidirectional image.

As shown in Figure 4a, the view angle $\left(\alpha ,\beta ,\varphi_{v},\varphi_{h}\right)$ of the virtual image was selected, where α is the horizontal azimuth. As shown in Figure 4b, where the coordinate of a pixel is m=[u,v], α is:

(3) $$\alpha =\mathrm{arcos}\frac{u}{\sqrt{u^{2}+v^{2}}}=\mathrm{arsin}\frac{v}{\sqrt{u^{2}+v^{2}}}\;$$

β is the vertical angle of the conventional camera. In our system, we set β as $90^{{}^{\circ}}$. Thus, the optical axis of the virtual image was perpendicular to the baseline defined by the omnidirectional and conventional cameras. In order to acquire a homogenous image pair, the resolution of the virtual perspective image w×h and the focal length f were set to be the same as parameters of the conventional image. $\varphi_{h}$ and $\varphi_{v}$ stand for the horizontal and vertical field of view, respectively, and can be calculated by:

(4) $$\varphi_{h}=\arctan\frac{w}{2f}\;$$

(5) $$\varphi_{v}=\arctan\frac{h}{2f}\;$$

Supposing that point M(i,j) in the virtual image plane under the three-dimensional coordinates can be expressed as $M^{\prime }=[i-W/2,H/2-j,0]$, it can be obtained by:

(6) $$M^{'}=M_{3}M_{2}M_{1}X_{s}\;$$

where $M_{1},M_{2},M_{3}$ are:

(7) $$M_{1}=\left[\begin{array}{cccc} \cos\alpha & -\sin\alpha & 0 & 0\\ \sin\alpha & \cos\alpha & 0 & 0\\ 0 & 0 & 1 & 0\\ 0 & 0 & 0 & 1 \end{array}\right],M_{2}=\left[\begin{array}{cccc} \cos\beta & 0 & -\sin\beta & 0\\ 0 & 1 & 0 & 0\\ \sin\alpha & 0 & \cos\beta & 0\\ 0 & 0 & 0 & 1 \end{array}\right],M_{3}=\left[\begin{array}{cccc} 1 & 0 & 0 & 0\\ 0 & 1 & 0 & 0\\ 0 & 0 & 1 & 0\\ 0 & 0 & 0 & f \end{array}\right]\;$$

**Figure 5:**
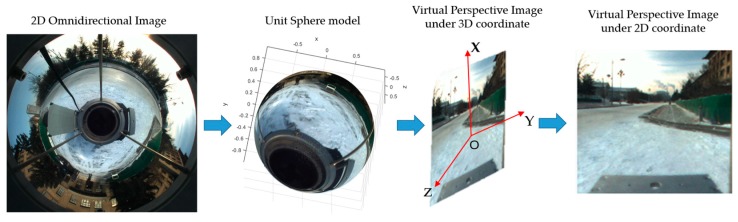
Overview of virtual perspective image generation.

Equation (6) establishes the one-to-one correspondence between $X_{s}$ and $M^{\prime }$. M[i,j] is the point in 2D coordinates, which can be derived from $M^{\prime }$ by simple coordinate transformation. An overview of virtual perspective image generation is shown in Figure 5. Thus, the virtual image which has the same intrinsic parameters with the conventional camera is derived.

### 4.2. Automatic Stereo Rectification

After obtaining the virtual perspective image, stereo rectification can be achieved by mapping the virtual and conventional images into the common plane and aligning the columns using rotation and translation matrices. These matrices can be obtained by decomposing the fundamental matrix. How to estimate the fundamental matrix robustly remains a challenging issue. Several methods for estimating the fundamental matrix have been proposed and can be classified into iterative and linear methods. Iterative methods [31,32] are more accurate than linear ones, but have high computational complexity and cannot eliminate the potential outliers. Linear methods contain the 7-point [33] and 8-point algorithm [34]. The main advantage of the 7-point algorithm is that a fundamental matrix can be estimated by using only seven points, but this fact becomes a drawback when some points are badly located or the corresponding points are redundant. The advantage of the 8-point algorithm is that it permits minimization of the error of estimating the fundamental matrix using redundant points. The algorithm is fast and easy to implement, but it is sensitive to noise and the solution is unstable. To improve the stability of the 8-point method, Harley et al. [29] normalized the corresponding points before using the 8-point method; in other words, they transformed the data into isotropy.

In our hybrid vision system, there is an affine transformation between the virtual image and the conventional image due to the different shooting angles of the omnidirectional camera and the conventional camera. Thus, ASIFT [28] was used to obtain the exact corresponding points between pairs of images because of its fully affine invariance. The 8-point algorithm with its normalization matrix [29] was adopted to obtain an accurate fundamental matrix through its speed and stability. One of the most important steps in our method was to optimize the normalization matrix. The following is an analysis of the epipolar geometry of the image pair and a detailed description of the optimization method for the normalization matrix.

#### 4.2.1. Epipolar Geometry Between Image Pairs

**Figure 6:**
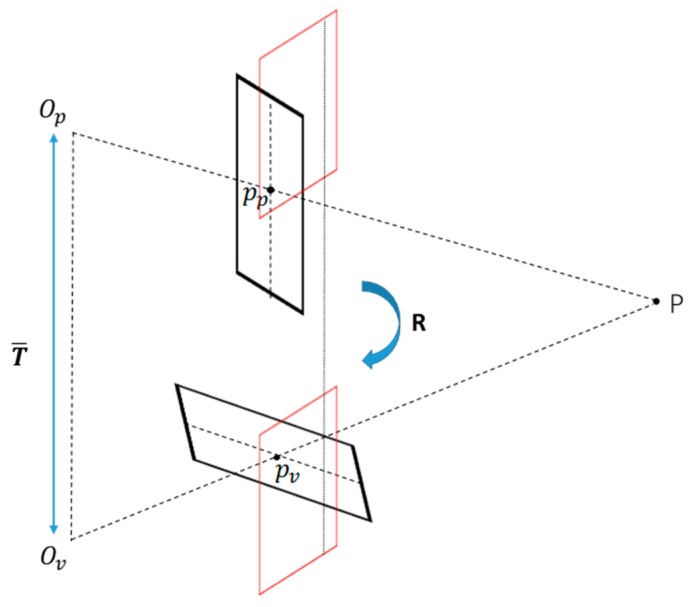
The epipolar geometry of the virtual perspective image and conventional image. $\pi_{p}^{\prime }$ and $\pi_{v}^{\prime }$ are the rectified images. It is obvious that the rectified images are column aligned.

As shown in Figure 6, $O_{p}$ and $O_{v}$ represent the projection center of the conventional camera and virtual perspective camera, respectively. The corresponding imaging planes are $\pi_{p}$ and $\pi_{v}$. p is a point in the three-dimensional coordinate. $p_{p}$ and $p_{v}$ are the point correspondence of P in the two image planes. The plane defined by $O_{p}$$O_{v}$ and p is the epipolar plane. The line defined by $O_{p}$ and $O_{v}$ is the baseline. According to [29], the relation of the image pixel coordinates and the fundamental matrix is:

(8) $$p_{p}^{T}Fp_{v}=0\;$$

The relation of the essential matrix and the fundamental matrix is:

(9) $$E=K_{p}^{T}FK_{v}\;$$

where $K_{v}$ and $K_{p}$ are the intrinsic parameters of the two images. The decomposition of an essential matrix is:

(10) $$E={\left[T\right]}_{\times }R\;$$

where $\bar{T}$ and T differ by a scale factor which can be calculated using two 3D points offline [35]. According to (9) and (10), (8) can be written as:

(11) $$p_{P}^{T}{\left(K_{P}^{T}\right)}^{-1}({\left[T\right]}_{\times }R)(K_{v}^{-1})p_{v}=0\;$$

Equation (11) establishes the relationship between corresponding points in the image pair and the rotation and translation matrices. Supposing that corresponding points are known, the translation matrix T and rotation matrix R can be calculated. Thus, the image planes $\pi_{p}$ and $\pi_{v}$ are rectified into $\pi_{p}^{\prime }$ and $\pi_{v}^{\prime }$ using T and R.

#### 4.2.2. Optimization Method of the Normalization Matrix

In order to improve the stability of the 8-point algorithm, raw data was transformed into isotropic data using normalization matrices [29]. However, the normalization matrix was calculated separately without considering the relative position of the image pair.

In our proposed method, we used a cost function to find the optimal normalization matrix and minimize the horizontal distance between corresponding pairs. The main processing algorithm is described in the following.

N pairs of correspondence points $\left(x_{i},x_{i}^{\prime }\right)$ i=1,2,⋯,N are obtained by ASIFT [28]. H and $H^{\prime }$ are two normalization matrices for two groups of points, respectively. The normalization can be achieved by:

(12) $${\bar{x}}_{i}=Hx_{i},{\bar{x}}_{i}^{\prime }=H^{\prime }x_{i}^{\prime }\;$$

where $\bar{x}$ and ${\bar{x}}^{\prime }$ are point correspondences after normalization. From (11) and (12), the following expression can be obtained:

(13) $$x_{i}^{T}H^{T}{\left(K_{P}^{T}\right)}^{-1}({\left[T\right]}_{\times }R)(K_{v}^{-1})H^{\prime }x_{i}^{\prime }=0\;$$

Thus, the error of epipolar geometry between two images can be expressed as:

(14) $$E(H,H^{\prime })=\sum_{i=1}^{N}x_{i}^{T}H^{T}{\left(K_{P}^{T}\right)}^{-1}({\left[T\right]}_{\times }R)(K_{v}^{-1})H^{\prime }x_{i}^{\prime }\;$$

In addition to the epipolar geometry constraint, the horizontal distance between two images is another significant factor. The horizontal distance between pair correspondences is:

(15) $$D(H,H^{\prime })=\sum_{i=1}^{N}\left(\left|\left(Hx_{i})-(H^{\prime }x_{i}^{\prime }\right)\right|\right)\;$$

According to (14) and (15), we define the objective function of the optimization problem as:

(16) $$S(H,H^{\prime })=\alpha E(H,H^{\prime })+\beta D(H,H^{\prime })\;$$

In order to minimize $S(H,H^{\prime })$, the iterative Expectation Maximization (EM) [36] is adopted because of its simplicity and effectiveness. α determines the ratio of epipolar geometry error and β the ratio of horizontal distance error. They are subject to α+β=1.

From (16), we can obtain the normalization matrices H and $H^{\prime }$. The corresponding points are normalized into isotropic points using H and $H^{\prime }$, which can obtain a more accurate result than the method proposed in [27].

## 5. Experimental Results and Analysis

### 5.1. Hybrid Stereo Vision System

**Figure 7:**
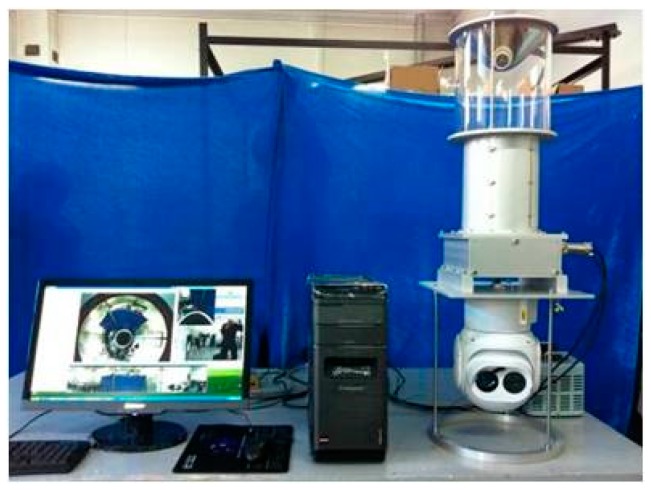
Experiment platform. The upper camera is omnidirectional, and the lower camera is conventional.

Figure 7 presents our experimental setup. Improved measurement accuracy can be achieved by adjusting the baseline distance. The hardware configuration of this experiment was a computer equipped with a dual-core Intel Pentium G2020 29 GHz, and 4 GB of RAM, running Windows 10. The system was implemented in VS2015 combined with OpenCV 2.4.9 and OpenGL 4.3. The cameras were synchronized via an external trigger. The parameters of the omnidirectional camera and the conventional camera are shown in Table 1. To verify the accuracy of the proposed rectification method, a stereo rectification experiment was performed.

### 5.2. Stereo Rectification Experiment with Real Image Pairs

**Figure 8:**
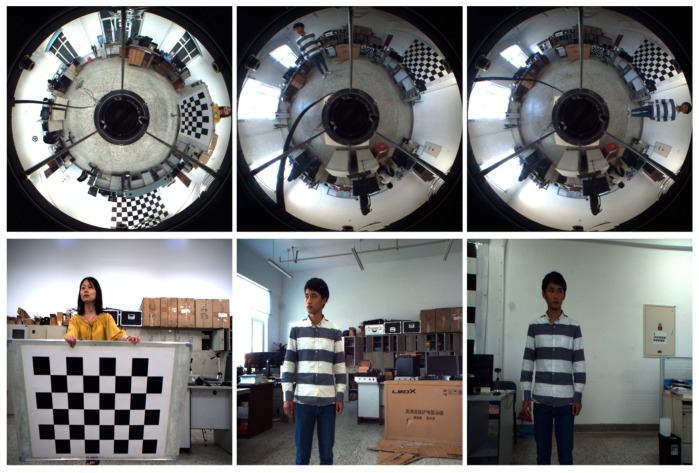
Three examples of the image pairs used for rectification accuracy comparison.

**Figure 9:**
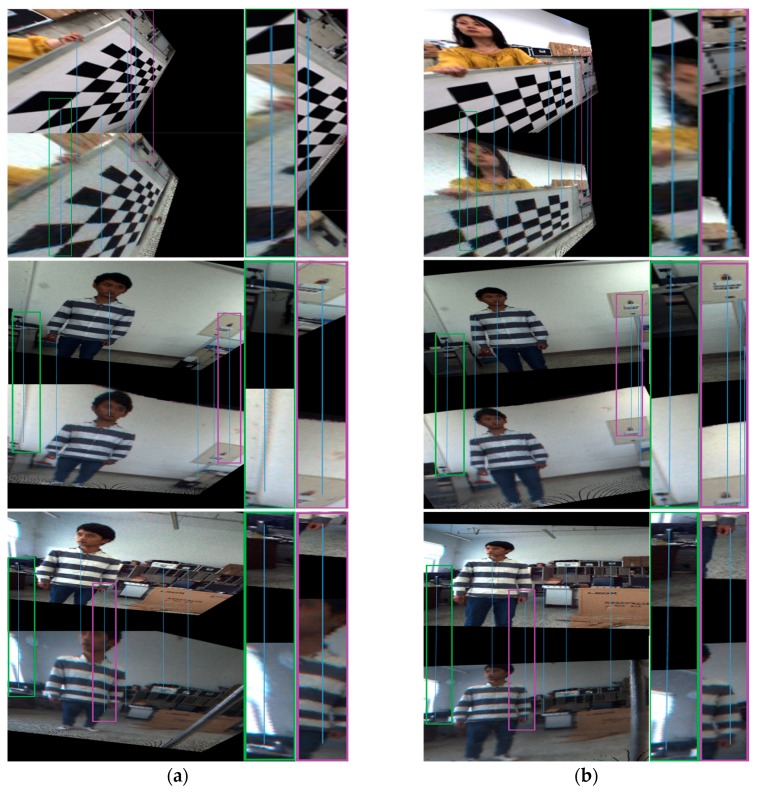
Stereo rectification results. The first row in each image is from the conventional camera. The second row in each image is from the omnidirectional camera. (**a**) The image pair with the rectification method in [27]; (**b**) the image pair with our proposed rectification method.

We first evaluated the performance of the proposed stereo rectification approach using 15 image pairs, where three of the original image pairs are shown in Figure 8. The results of the three image pairs with the rectification algorithm from [27] and with the proposed rectification approach are shown in Figure 9a,b. As can be seen from these three pairs of images, the performance improvement obtained by the proposed solution is evident. To show the accuracy of the rectification, we highlighted a few notable regions where results of the rectification method from [27] exhibited misalignments, whereas our results remain aligned in these regions. In addition, it is obvious that the distortion at the edge in Figure 9a is not calibrated with the method proposed in [27].

To represent rectification error quantitatively, we selected four stereo image pairs from the indoor environment that were rectified using the rectification method from [27] and our proposed method. From each image set, we randomly selected 30 corresponding corners and calculated the mean deviation of the horizontal distances. We summarized the average difference of each individual image set along with their overall average (term Average Err.). Table 2 depicts these results in pixels. From the average error, we can calculate that the accuracy of our method increased by 34.78% compared with the method from [27]. Since rectification gives a pair of images in which corresponding epipolar lines should be collinear and parallel to the vertical axis, the abovementioned criterion is suitable for computing the error in a rectified pair of images.

### 5.3. Odometry in a Simulated Environment

**Figure 10:**
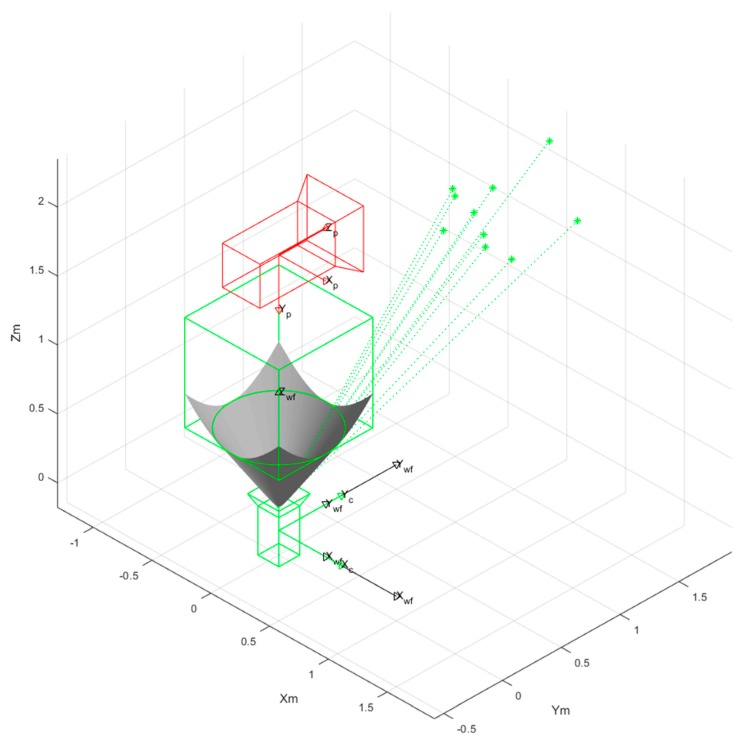
A simulated environment with one omnidirectional image and one conventional image.

**Figure 11:**
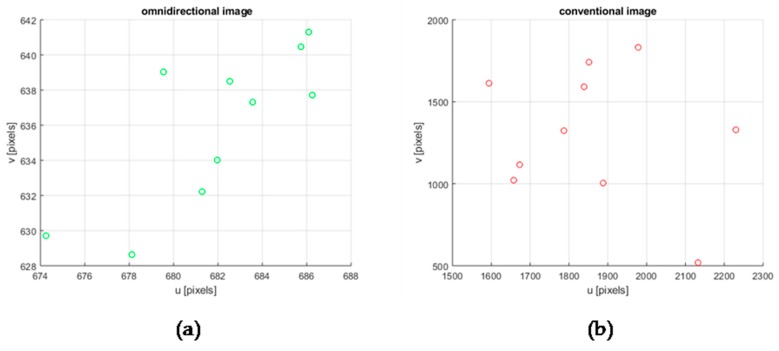
Sample omnidirectional (**a**) and perspective (**b**) images captured in the simulated environment.

**Figure 12:**
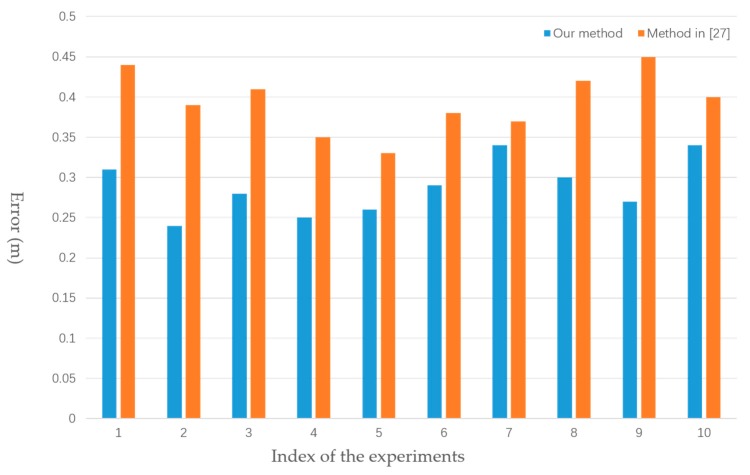
Mean errors of ten experiments with different orientation angles.

Stereo vision odometry is based on the parallax of two images. The accuracy of odometry represents the accuracy of the rectification method. Thus, we analyzed odometry accuracy in a simulated environment. As shown in Figure 10, we placed the omnidirectional camera at the origin. In other words, the camera coordinate was consistent with the world coordinate, while the conventional camera was put at (0,0,2). The parameters of the cameras were set to the same values as in Table 1. We performed ten groups of experiments where each had a different rotation and translation matrix. We placed ten points in 3D coordinates for each experiment. Captured points of one experiment in an omnidirectional image and perspective image are shown in Figure 11a,b, respectively. Ten points were used to calculate the R and T matrixes. The image pairs were rectified using our proposed approach and the method in [27], respectively. The vertical disparities $Y_{2}-Y_{1}$ of 10 corresponding points were derived. The distances between sample points and cameras were calculated by (17). f is the camera focal length, and L is the length of the vertical baseline. In our simulated experiment, f=310.57 mm, L=345.584 mm. The value of the *Y*-axis of each point was used as a ground truth. The mean errors of distance in different orientations between cameras are shown in Figure 12. Compared with the method in [27], the mean error in each experiment decreased by 0.1–0.2 m using our proposed approach. It can easily be inferred that our proposed rectification approach is more reliable.

(17) $$d_{i}=\frac{fL}{Y_{2}-Y_{1}}\;$$

### 5.4. Real-Time Target Tracking and Odometry Experiment

To illustrate the performance of the proposed rectification method and provide a direct application, we used the hybrid vision system to track a target with a size of 1.7 × 0.6 × 0.3 m and perform odometry. The tracking algorithm for the omnidirectional camera in [37] was adopted. The cameras shown in Figure 7 was placed in a fixed position. The target tracking and odometry real-time experiment had a total output of 154 frames over a period of 20 s, while the average computation time of each frame was 179.87 ms.

**Figure 13:**
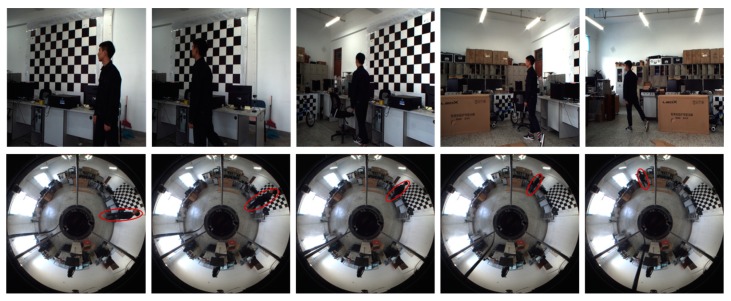
The tracking and cooperation result of the two cameras. From left to right, the 17th, 26th, 35th, 43rd, and 85th frame are shown.

Five frames were randomly selected from the processing results, and are shown in Figure 13. It demonstrates that the angle from the omnidirectional camera can be sent to the conventional camera successfully, and the two cameras can cooperate with each other very well.

**Figure 14:**
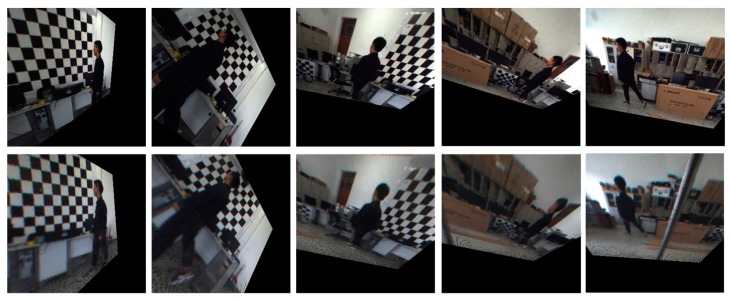
The rectification result of image pairs in Figure 13.

**Figure 15:**
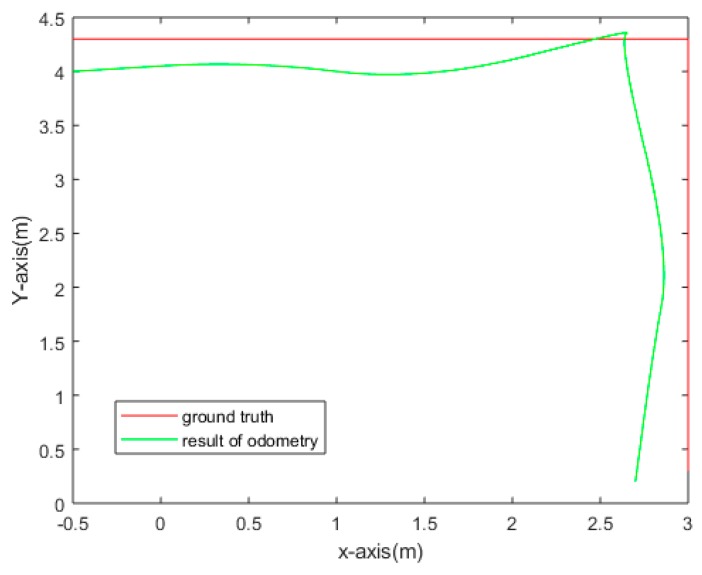
The comparison of odometry results and ground truth.

We used our proposed method to rectify image pairs, the results of which are shown in Figure 14. The comparison of the odometry results and the ground truth is shown in Figure 15 (the hybrid vision system was fixed at the origin). The average error distance is 0.317 m. The experimental results show that the error is far less than the target scale. We can conclude that the calculated trajectory is consistent with the ground truth, and our hybrid vision system can be used in surveillance tasks.

## 6. Discussion

In summary, we demonstrated an automatic rectification approach for the hybrid vision system. The geometric relationship between omnidirectional and conventional images was simplified by generating a virtual perspective image from the omnidirectional image. Image pairs were rectified using the 8-point algorithm with an optimized normalization matrix. We showed that the row coordinate parallax of rectified image pairs was within 2 pixels. The mean errors of the odometry based on triangulation were less than 12%. Based on this, we consider that the error of rectification is acceptable when compared with other methods in literature. Deng et al. [20] also used the corresponding points to acquire the extrinsic parameters of the hybrid vision system, but they used 3D points instead of 2D points on the plane. 3D points were extracted based on 3D Euclidean reconstruction of scene points, which involves high computational complexity due to the establishment of the polynomial approximation model. Lin et al. [27] also generated a virtual perspective image to simplify the epipolar geometry between hybrid cameras, but they synthesized the virtual plane by back-projecting the rays directly from the omnidirectional image. This also suffers from calculation complexity due to the non-linear imaging model of the omnidirectional image. In addition, they derived a 3D point by calculating the intersection of two rays, which cannot obtain an accurate result. Among the previously proposed practical solutions for using an omnidirectional–conventional camera pair, only one of them actually estimates the relative position and orientation of the cameras, which is given in [27]. Therefore, we numerically compared the accuracy of the parameter estimation of our method only with [27].

It is noteworthy that the number of corresponding points strongly affects the rectification performance, because the fundamental matrix is estimated based on their position. The experiment in no salient feature scene was performed. The error of the fundamental matrix was very large since almost no corresponding points were detected.

In the future, we would like to use our approach for different camera combinations, including omnidirectional-fisheye and fisheye-conventional. Additionally, we plan to develop an improved method which is not limited by various features in the scene.

## 7. Conclusions

In this paper, an active hybrid vision system consisting of an omnidirectional camera and a conventional camera was presented. We provided the key techniques to rectify image pairs automatically. The virtual perspective image from an omnidirectional image was obtained using the proposed perspective projection model. ASIFT and the 8-point algorithm with an optimized normalization matrix were applied to rectify the image pair automatically. Our investigation in the simulated and real environment has demonstrated that the proposed approach not only overcomes the shortcomings of high computational complexity in the hybrid vision system, but is superior to other state-of-the-art methods [27] in regard to accuracy. Therefore, our system can effectively meet the requirements of vision sensors in surveillance tasks.

## Figures and Tables

**Table 1:** Camera parameters given by the manufacturer.

Hyperbolic Mirror Parameters		Omnidirectional Camera Parameters		Conventional Camera Parameters	
a (Major axis)	31.2888 mm	Part Number	FL2G-50S5C-C	Part Number	FL2G-50S5C-C
b (Minor axis)	51.1958 mm	Resolution	1360 × 1360 pixels	Resolution	2448 × 2048 pixels
mapping parameter	0.82	Frame rate	10 frames/s	Frame rate	10 frames/s
vertical viewing angle	120°	Interface	1394 b	Interface	1394 b

**Table 2:** Comparisons of rectification errors for 4 rectified stereo image pairs (in pixels).

	Method in [27]	Our Proposed Method
Set 1	2.457	1.401
Set 2	2.374	1.645
Set 3	2.621	1.831
Set 4	1.987	1.176
Average Err.	2.360	1.513
